# Similarities and Differences of the Soleus and Gastrocnemius H-reflexes during Varied Body Postures, Foot Positions, and Muscle Function: Multifactor Designs for Repeated Measures

**DOI:** 10.1186/1471-2377-11-65

**Published:** 2011-06-02

**Authors:** Hesham N Alrowayeh, Mohamed A Sabbahi, Bruce Etnyre

**Affiliations:** 1 Kuwait University, Faculty of Allied Health Sciences, Physical Therapy Department, State of Kuwait; 2 Texas Woman's University, School of Physical Therapy, Houston, Texas, USA; 3 Rice University, Kinesiology Department, Houston, Texas, USA

## Abstract

**Background:**

Although the soleus (Sol), medial gastrocnemius (MG), and lateral gastrocnemius (LG) muscles differ in function, composition, and innervations, it is a common practice is to investigate them as single H-reflex recording. The purpose of this study was to compare H-reflex recordings between these three sections of the triceps surae muscle group of healthy participants while lying and standing during three different ankle positions.

**Methods:**

The Sol, MG and LG muscles' H-reflexes were recorded from ten participants during prone lying and standing with the ankle in neutral, maximum dorsiflexion, and maximum plantarflexion positions. Four traces were averaged for each combination of conditions. Three-way ANOVAs (posture X ankle position X muscle) with planned comparisons were used for statistical comparisons.

**Results:**

Although the H-reflex in the three muscle sections differed in latency and amplitude, its dependency on posture and ankle position was similar. The H-reflex amplitudes and maximum H-reflex to M-response (H/M) ratios were significantly 1) lower during standing compared to lying with the ankle in neutral, 2) greater during standing with the ankle in plantarflexion compared to neutral, and 3) less with the ankle in dorsiflexion compared to neutral during lying and standing for all muscles (*p *≤ .05).

**Conclusion:**

Varying demands are required for muscles activated during distinctly different postures and ankle movement tasks.

## Background

Hoffmann reflex (H-reflex) recordings from muscles around the ankle joint are useful in examining the activation and inhibition during normal function. It is also useful for examining dysfunctional pathologies. For example, H-reflex recordings from the soleus (Sol) muscle are useful electrophysiological procedures for the evaluation of patients with S1 radiculopathy [1,2].

Existing H-reflex procedures do not allow valid examination of the L5 nerve root. Clinicians either measure the L4 and L5 nerve roots by recording the vastus medialis H-reflex or measure the L5 and S1 nerve roots by recording the Sol H-reflex. Then inference is used to diagnosis L5 root impingement with a corroborating physical exam and/or radiographic imaging.

H-reflex recordings from the lateral gastrocnemius (LG) would provide L5 diagnosis in patients with radiculopathy, given that the LG is innervated by L5 more than S1 nerve roots and the Sol is innervated mostly by the S1 nerve root [3]. Thus, it may be possible to detect S1 radiculopathies using the Sol H-reflex and L5 radiculopathies from LG H-reflex recordings. However, these cannot be valid measurements until normal activation patterns under varied recordings condition are established.

No previous study has compared H-reflex recordings between the LG, medial gastrocnemius (MG), and Sol during varied body postures, foot positions, and muscle function. Yet, it is commonly accepted that the Sol, LG and MG which function as members of the triceps surae muscle group have similar activation patterns, although they perform different functions [4], differ in structure and muscle fiber types [5,6], and are innervated differently [3]. Such differences in anatomic and physiologic functions between the two muscle groups/components result in different activation patterns. Thus, it may be possible to record differences in the reflex responses of the Sol, MG, and LG muscles during varied body postures, ankle positions, and functional muscle activities from clinical or laboratory measurements. The purpose of this study was to compare the latencies and amplitudes of H-reflex recordings from the Sol, MG and LG in healthy individuals during varied recording conditions (i.e. lying or standing with ankle dorsiflexion, plantarflexion, or neutral). This may be particularly important because previous published reports found the H-reflex changes with varied body postures and joint positions [7-14]. Results of this study may provide clinicians and researchers with 1) an understanding of the normal H-reflex activation patterns recorded from muscles around the ankle joint, and 2) a reference standard for comparison to patients with L5 and/or S1 nerve root impingements.

## Methods

### Participants

Ten healthy males (mean age = 32.3 ± 6.5 years, height = 176.1 ± 9.8 cm, and weight = 84.0 ± 11.1 kg) participated in this study. None of the participants reported any history of musculoskeletal, metabolic, systemic, or neurologic disorders within the past two years. All participants read and signed an informed consent approved by the Institutional Ethical Review Board.

### H-reflex stimulation and recording

The MG, LG, and Sol H-reflexes were stimulated and recorded according to the method of Sabaahi and Khalil [15] and Alrowayeh and Sabbahi [16]. The reliability of H-reflex recordings during varied postures and ankle position were previously established for the MG (ICC = 0.63 to 0.94) and LG (ICC = 0.58 to 0.94) [16]. For the Sol, the ICC reliability was 0.8 during supine lying and it was 0.93 during standing [17].

**Figure 1:**
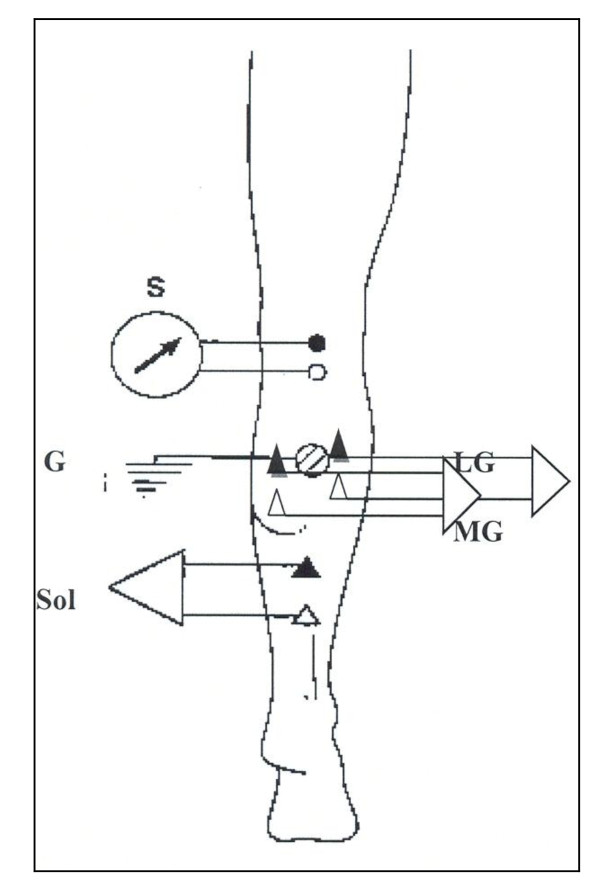
**Location of the stimulating and recording electrodes for the Sol, MG, and LG H-reflexes**. (S) Stimulus, Sol recording, MG recording, LG recording, and (G) ground electrodes. The small circles represent the positions of the electrode for electrical stimulation and the triangles represent the positioning of the recording electrodes.

In brief, an EMG (Cadwell Laboratories, Inc., Kennewick, WA) unit (set at a gain of 1000x to 5000x, a filter bandpass of 10 Hz to 10 kHz, sampling rate of 78.6 KHz, and input range of data acquisition of +/- 10 V) electrically stimulated and recorded the MG, LG, and Sol H-reflexes. To stimulate the H-reflexes, one Ag/AgCl surface stimulating bar electrode was applied over the tibial nerve longitudinally at the midline of the popliteal fossa with the active electrode positioned proximal to the reference electrode to avoid anodal block [18] (Figure 1). The stimulating electrode delivered focal percutanous electrical stimuli of 1.0 ms square-wave pulses. This single stimulation point elicited the H-reflexes in all three muscles (Sol, MG and LG) simultaneously. The stimulation intensity was increased by 2 mA increments from zero until maximal M-wave was obtained to map the amplitude of the H-reflex and muscle action recruitment curve. To record the H-reflex amplitude and M-wave, two Ag/AgCl surface recording bar electrodes (distance between electrode leads was 2.5 cm) were attached over the bellies of the MG and LG muscles, distal to the motor innervation point. For the Sol muscle, a third bar electrode was attached approximately 8 cm distal to the distal point of intersection of the two heads of gastrocnemii in line with the Achilles tendon (Figure 1). The recording bar electrodes were placed so that the reference electrode was distal to the active electrode. One 2-cm diameter round metal ground electrode was placed over the proximal body of the MG and LG, between the recording and the stimulating electrodes (Figure 1). Throughout the procedures, electrodes were maintained in the same positions.

### Experimental procedures

The skin was prepared for stimulation and recording by abrading with fine sandpaper and cleaning with alcohol. Electrodes with conductive gel were then applied to the appropriate locations and secured in placed with adhesive tape for the duration of the recording session.

After placement of electrodes, each participant's Sol, MG, and LG H-reflex recruitment curves were recorded during prone lying and standing postures. In the prone lying position, the participant was asked to lie in a prone position while maintaining the ankle in a neutral (90 degrees of foot to shank angle), maximum active dorsiflexion, or maximum active plantarflexion position over the end of a treatment table. In the standing position, each participant was asked to perform each of three standing conditions: standing upright relaxed (ankle in the neutral position, 90 degrees of foot to shank angle); standing on heels (maximum active dorsiflexion); or standing on metatarsals (maximum active plantarflexion) while maintaining equal weight on both lower extremities. Maximum muscle activation during plantarflexion and dorsiflexion while lying and standing was used to simulate strenuous activities of the feet during daily living activities. The orders of recordings from the muscles, postures and ankle positions were randomly alternated to compensate for the potential inherent changes of the H-max recording during the course of the experiment [19]. Five traces were elicited and recorded for each participant at each incremented electrical stimulus. The largest four traces were included in the analysis.

### Signal and data analyses

The maximum peak-to-peak amplitude and latency of the largest four traces from the Sol, MG, and LG H-reflexes were measured and averaged for each testing position. Also, the peak-to-peak amplitude of the maximum M-wave was measured. Then, the H/M (maximum H-reflex to maximum M-response) ratio was calculated for each muscle/posture/ankle-position condition.

Means and standard deviations for the Sol, MG, and LG H-reflex maximum amplitudes, latencies, and H/M ratios for each condition were calculated over subjects. Statistical analysis included two dependent variables (H-reflex maximum amplitude and H/M ratio) and three independent variables: 1) muscles (Sol, MG, and LG); 2) ankle positions (neutral, dorsiflexion, plantarflexion); and 3) body postures (prone lying and standing). A 3 × 3 × 2 (muscles X ankle positions X body postures) repeated measures ANOVA with planned comparisons was performed with a global alpha level of .05. Planned comparisons included the mean differences of H-reflex maximum amplitudes and H/M ratios recorded from each muscle during prone lying and standing in dorsiflexion and plantarflexion positions to the mean values when the ankle was in the neutral position. Planned comparisons of H-reflex means were also made among muscles during prone lying and standing with the ankle in the neutral position. Statistical analyses were conducted using SPSS (Version 16, SPSS Inc., Chicago, IL).

## Results

**Figure 2:**
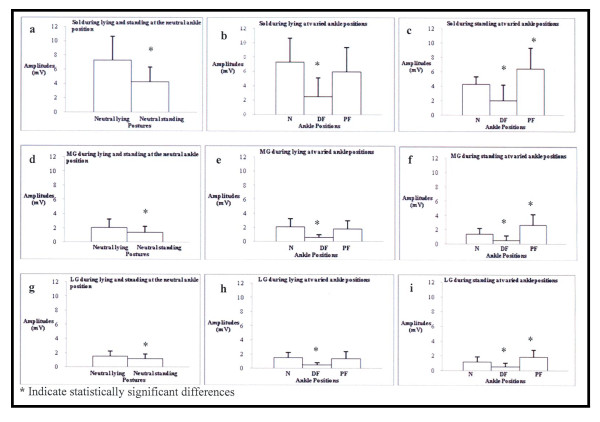
**Comparisons of H-reflex means and standard deviations amplitudes for the Sol, MG, and LG during lying or standing with the ankle in neutral (N), dorsiflexion (DF), and plantarflexion (PF)**.

Planned comparisons showed the Sol, MG, and LG H-reflex maximum amplitudes means were significantly less during standing upright compared to prone lying with the ankle in the neutral position (*p *= .006, .009, and .029; Figure 2a, d, g). The planned comparisons also showed the average Sol, MG, and LG H-reflex maximum amplitudes during prone lying or standing were significantly less during the ankle dorsiflexion compared to the neutral ankle position (*p *= .0005 and .007 for Sol, Figure 2b, c; *p *= .001 and .04 for MG, Figure 2e, f; and *p *= .0005 and .0005 for LG, Figure 2h, i), the average Sol, MG, and LG maximum amplitudes did not significantly change for ankle plantarflexion compared to the neutral ankle position during prone lying (*p *= .2, .19, and .47), and the Sol, MG, and LG maximum amplitude was significantly greater during ankle plantarflexion compared to the neutral ankle position during the standing posture (*p *= .005, .024. and .01). The results for the Sol, MG, and LG H-reflex H/M ratio were comparable to the H-reflex maximum amplitude results during prone lying or standing for all ankle positions (Table 1).

**Table 1:** Means and standard deviations (below the means in parentheses) for the Sol, MG, and LG H-reflex maximum amplitudes in millivolts (mV), latency (ms), and H/M ratio during lying and standing with the ankle in neutral (N), dorsiflexion (DF), and plantarflexion (PF)

H-reflex parameters	Ankle Positions	Muscle and posture					
		
		Sol		MG		LG	
		
		Lying	Standing	Lying	Standing	Lying	Standing
**Amplitude**	**N**	7.25*† (3.35)	4.28*§‡ (2.03)	2.06*† (1.20)	1.38*§‡ (0.85)	1.50*† (0.70)	1.15*§‡ (0.67)
	
	**DF**	2.47† (2.63)	1.96§ (2.18)	0.60† (0.39)	0.53§ (0.60)	0.47† (0.34)	0.49§ (0.51)
	
	**PF**	5.95 (3.37)	6.40‡ (2.88)	1.76 (1.19)	2.65‡ (1.52)	1.38 (0.89)	1.81‡ (0.97)

**H/M**	**N**	0.51*† (0.17)	0.38*§‡ (0.19)	0.42*† (0.30)	0.20*§‡ (0.11)	0.28*† (0.13)	0.18*§‡ (0.08)
	
	**DF**	0.22† (0.18)	0.19§ (0.18)	0.17† (0.14)	0.08§ (0.08)	0.18† (0.04)	0.11§ (0.12)
	
	**PF**	0.40 (0.24)	0.60‡ (0.27)	0.32 (0.25)	0.39‡ (0.15)	0.22 (0.09)	0.32‡ (0.14)

**Latency**	**N**	30.2 (2.3)	30.6 (2.3)	29.3 (2.2)	29.3 (2.3)	29.1 (2.1)	28.9 (2.4)
	
	**DF**	30.4 (2.5)	30.9 (2.3)	29.3 (2.3)	29.3 (2.3)	29.0 (2.3)	29.0 (2.6)
	
	**PF**	30.5 (2.3)	30.8 (2.3)	29.1 (2.2)	29.1 (2.4)	28.8 (2.4)	28.7 (2.1)

* indicates significant differences between lying and standing for each muscle with the ankle in neutral position for both amplitude and H/M ratio parameters.

† indicates significant differences between dorsiflexion and neutral position for each muscle while lying for both amplitude and H/M ratio parameters.

§ indicates significant differences between dorsiflexion and neutral position for each muscle while standing for both amplitude and H/M ratio parameters.

‡ indicates a significant difference between plantarflexion and neutral position for each muscle while standing for both amplitude and H/M ratio parameters.

**Figure 3:**
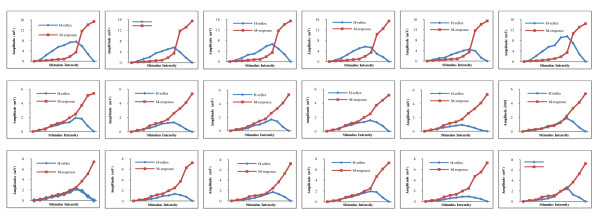
**Recruitment curves for a participant**. The Sol (raw 1), MG (raw 2), and LG (raw 3) muscles during the lying posture with the ankle in the neutral (column 1), dorsiflexion (column 2), or plantarflexion (column 3) positions and during the standing posture with the ankle in the neutral (column 4), dorsiflexion (column 5), or plantarflexion positions (column 6). Horizontal axis which is the stimulus intensity (straight lines). Vertical axis which is the H-reflex amplitude (bell curves).

Results showed the Sol H-reflex was recorded at sub-threshold to the M-response whereas MG and LG H-reflexes were supra-threshold to the M-response in most of the participants (Figure 3). The average maximum peak-to-peak amplitude of the Sol H-reflex during prone lying or standing was greater than the average maximum peak-to-peak amplitudes for the MG and LG H-reflex measures for all ankle positions. The differences were statistically significant (Table 1).

Table 1 also shows the latencies for the MG and LG during prone lying or standing were earlier than the Sol H-reflex latency for all ankle positions. The latency ranged from 29.1 to 29.3 ms for the MG, from 28.7 to 29.1 ms for the LG, and from 30.2 to 30.9 ms for Sol H-reflexes. The latency was significantly different between the Sol and MG (*p *= .006), Sol and LG (*p *= .0005), but not between the MG and LG (*p *= .49) across all ankle positions and both postures.

## Discussion

The two primary results of this study showed: 1) The Sol, MG, and LG H-reflexes during prone lying and standing for three active ankle positions were similar in some respects. The Sol, MG, and LG H-reflex maximum amplitudes were inhibited during standing compared to lying with the ankle in the neutral position, inhibited during dorsiflexion compared to the neutral ankle position during lying and standing, and facilitated during plantarflexion compared to the neutral ankle position while standing; and 2) The Sol, MG, and LG muscle activities differed in terms of reflex recruitment (peak-to-peak amplitudes and thresholds of stimulation).

### Similarities between the Sol, MG, and LG H-reflex recordings

The similarities between the muscles (Sol, MG, and LG) H-reflex average maximum amplitudes, particularly during standing, may be explained by their role as antigravity postural muscles in which the suppression of these muscles would prevent body instability (swaying) and retain balance necessary for upright posture. Similarities may also be explained by how these muscles perform during locomotion [20].

The suppression of the Sol, MG, and LG H-reflex maximum amplitudes during standing upright may be due to presynaptic inhibition, in which somatosensory afferents from the foot sole and/or stretch receptors from the soleus muscle may have been the source of presynaptic inputs. This was previously reported as a possible mechanism for this unintuitive result of greater H-reflex amplitudes during prone lying compared with active standing [7,8,10-14]. Reciprocal inhibition may also have contributed to the recorded suppression of the Sol, MG, and LG H-reflex maximum amplitudes. This was more likely during active ankle dorsiflexion while lying or standing, when the contraction of antagonistic muscles (i.e., anterior tibialis) may have evoked reciprocal Ia inhibition on the triceps surae muscles [21], resulting in the observed average Sol, MG, and LG H-reflex maximum amplitude suppression. Another inhibitory mechanism which may have contributed to the recorded suppression of the Sol, MG, and LG H-reflex maximum amplitudes is vestibular inhibition [22]. In this study, participants' H-reflexes were measured after stabilizing standing posture to avoid the vestibular effect on the H-reflex measurements due to dynamic postural changes. Therefore vestibular inhibition is an unlikely explanation for these results, but may have contributed. These mechanisms cannot be distinguished in this study, as it is possible all three inhibitory mechanisms (i.e., presynaptic, reciprocal, and vestibular influences) may have contributed to the H-reflex suppression among the Sol, MG, and LG with varying amounts of inhibition.

The facilitation of the Sol, MG, and LG H-reflexes during standing on metatarsal (maximum plantarflexion) may be explained by the increase in the excitability drive to the alpha motoneurons of the Sol, MG, and LG muscles as subjects contracted these muscles against gravity on the body's mass [23]. By comparison, in the present study there were no changes for the Sol, MG and LG H-reflex maximum amplitudes during prone lying with ankle in plantarflexion. The likely explanation for this is the greatly reduced requirement of excitability drive to the alpha motoneurons of the Sol, MG, and LG muscles as they were only displacing the foot segment against gravity rather than the entire body mass.

The inhibition of the Sol and MG H-reflexes during ankle dorsiflexion and facilitation during ankle plantarflexion reported in this study were in agreement with a previously reported study [24]. Pinniger and colleagues [24] reported Sol and MG H-reflexes were inhibited during passive lengthening and facilitated during passive shortening actions. The current study extends those observations for the Sol and MG inhibition and facilitation during active lengthening and shortening, respectively. The inhibition and facilitation of the Sol and gastrocnemius H-reflexes under similar conditions of static postures and ankle positions reported in this study, however, were in conflict with previously reported results [25]. Moritani and colleagues [25] found differences between the Sol and MG H-reflex amplitudes (i.e., facilitation of the gastrocnemius and inhibition of the Sol) during the dynamic functional performance during hopping. Differences between the Moritani, et al. study [25] and the present findings may be explained in part by the differences between the dynamic and static functional movements during H-reflex measurements. In this study, participants were either statically lying or standing while maintaining the three ankle positions.

Although the major goal of this study was to establish the normal activation patterns of the muscles around the ankle joint under varied recordings condition, the study findings may be of clinical relevance. It provides a reference standard for comparison of patients with L5 nerve root impingement, as existing H-reflex procedures do not allow valid examination of the L5 nerve root. However, the validation of the clinical diagnosis of L5 and S1 radiculopathies requires patients with proven radiculopathies at different levels and it is the focus of ongoing research. Nevertheless, testing patients with proven radiculopathies using the H-reflex has been shown to be more useful during standing as compared to lying. It increases the sensitivity of the H-reflex for detecting subtle changes [11]. Thus, future studies of patients with L5/S1 radiculopathies may find it beneficial to use this method of examining H-reflexes for differentiating L5 and S1 impingements.

### Differences in motoneuron recruitment between the Sol, MG, and LG H-reflexes

The differences between the Sol, MG, and LG H-reflex maximum peak-to-peak amplitudes and H/M ratios reported in this study were in agreement with the work of Tucker and Türker [26]. The differences of maximum peak-to-peak amplitudes may be due to motoneuron recruitment tendency differences, with the Sol motoneurons having greater recruitment tendencies from the influence of muscle spindles than the MG and LG muscles because Sol motoneurons have a lower recruitment threshold [26]. This was supported by the present study results, in which the Sol H-reflex was mostly recorded at subthreshold maximum M-wave levels to muscle action potential than those of the MG and LG muscles (Figure 3). Threshold differences may be due to the tonic characteristics of the Sol muscle which have a greater percentage of muscle spindle afferents than gastrocnemius muscle heads. The geometry of Ia afferent fibers' locations in the tibial nerve supplying the Sol, MG, or LG could be another factor contributing to reflex recruitment differences. Ia afferents originating from the Sol muscle may be in the more superficial fascicles of the tibial nerve. This may facilitate the elicitation of greater H-reflex amplitudes and help explain the lower threshold.

## Limitations

In this study, tibialis anterior muscle activity was not monitored before or during active ankle movements while prone lying and standing. Previous research showed co-contraction of agonist and antagonist muscles depressed the H-reflex amplitudes [21]. The present findings, thus, may have some methodological limitation, although the co-contraction of the tibialis anterior and triceps surae muscles during neutral and plantarflexion ankle positions in prone lying and standing was shown to be minimal [27]. It is possible that cross talk may have contributed to the recorded H-reflex. However, because we used bipolar recording electrodes, kept a distance between the three electrodes and maintained the orientation of the active and reference electrodes, cross talk was reduced. External validity is also limited because results were based on 10 participants.

## Conclusion

The results of this study suggested the varying demands for static/tonic standing requires multiple physiologic responses around the ankle which are performed by the multi-task triceps surae muscle group. The results also suggested the sensitivity of H-reflex procedures to detect subtle changes in physiologic activity of functionally closely related muscles (Sol, MG, and LG) bundled in one compartment group (triceps surae) but perform contractions across the multiaxial ankle joint. The results of this study provide a reference standard for comparison of patients with nerve root impingement at the L5 and S1 levels. Future studies of patients with L5/S1 radiculopathies may find it beneficial to use this method of examining Sol, MG, and LG H-reflexes during plantarflexion, neutral and dorsiflexion standing for differentiating L5 and S1 impingements.

## Competing interests

The authors declare that they have no competing interests.

## Authors' contributions

HNM contributed to the following activities: concept and research design, writing, project management, and data collection and analysis. MAS contributed to the following activities: concept and research design, and writing. BE contributed to writing and consultation. All authors read and approved the final manuscript.

## Pre-publication history

The pre-publication history for this paper can be accessed here:

http://www.biomedcentral.com/1471-2377/11/65/prepub
